# Factors associated with childhood diarrheal in Ethiopia; a multilevel analysis

**DOI:** 10.1186/s13690-021-00566-8

**Published:** 2021-07-06

**Authors:** Setegn Muche Fenta, Teshager Zerihun Nigussie

**Affiliations:** Department of statistics, Faculty of Natural and Computational Sciences, Debre Tabor University, Debra Tabor, Ethiopia

**Keywords:** Diarrhea, Under-five children, Multilevel analysis, Ethiopia

## Abstract

**Background:**

Diarrhea is the second cause of child deaths globally. According to World Health Organization reports, in each year it kills more than 525,000 children under-5 years. More than half of these deaths occur in five countries including Ethiopia. This study aimed to identify both individual and community-level risk factors of childhood diarrheal in Ethiopia.

**Methods:**

Ethiopian demography and health survey of 2016 data were used for the analysis. A total of 10,641 children aged 0–59 months were included in the analysis. A multi-level mixed-effect logistic regression model was used to identify both individual and community-level risk factors associated with childhood diarrheal.

**Result:**

The incidence of childhood diarrheal was 12% (95%CI: 11.39, 12.63). The random effect model revealed that 67% of the variability of childhood diarrhea explained by individual and community level factors. From the individual-level factors, children aged 36–59 month (AOR = 3.166; 95% CI: 2.569, 3.900), twin child (AOR = 1.871; 95% CI: 1.390, 2.527), birth order 5 and above (AOR = 2.210, 95% CI: 1.721, 2.839), not received any vaccination (AOR = 1.197; 95% CI: 1.190,1.527), smaller size of child at birth (AOR = 1.303;95% CI: 1.130,1.504) and never breastfed children (AOR = 2.91;95%CI:2.380,3.567) associated with the higher incidence of childhood diarrhea. From the community-level factors, living in a rural area ((AOR = 1.505; 95%CI: 1.233, 1.836)), unprotected source of drinking water (AOR: 1.289; 95% CI: 1.060, 1.567) and availability of unimproved latrine facilities (OR: 1.289; 95% CI: 1.239, 1.759) associated with the higher incidence of childhood diarrhea. Besides, Children live in Afar, Amhara, Benishangul-Gumuz, Gambella, SNNPR and Dire Dawa regions had higher incidence of childhood diarrhea**.**

**Conclusion:**

The incidence of childhood diarrhea was different from cluster to clusters in Ethiopia. Therefore, integrated child health intervention programs including provisions of toilet facility, access to a clean source of drinking water, educate parents about the importance of breastfeeding and vaccination have to be strongly implemented in order to reduce the high incidence of childhood diarrhea among children in Ethiopia.

## Background

Diarrhea is defined as the passage of three or more loose or liquid stools per day (or more frequent passage than is normal for the individual). It is usually a symptom of gastrointestinal infection, which can be caused by a variety of bacterial, viral, and parasitic organisms. Infection is spread through contaminated food or drinking-water, or from person to person as a result of poor hygiene [1, 2].

Diarrhea is the second most common cause of child deaths and the leading cause of malnutrition in children under 5 years old. According to World Health Organization reports, in each year it kills more than 525,000 children under-5 years, approximately 1439 every day. Fifty percent of the deaths occurred in five countries including Ethiopia. Worldwide, more than 1.7 billion cases of childhood diarrheal disease occurred in every year. For instance, about 80% of morbidity and mortality occur in sub-Saharan Africa and South Asia. Malnutrition is an underlying factor in one third of preventable child deaths and plays a significant role in fatal diarrhea episodes [1, 2].

Ethiopia is second country in Africa with the highest number of diarrheal deaths. Diarrhea is the second leading root of death and contributes to more than one in every ten child deaths in Ethiopia [3, 4]. The incidence of childhood diarrhea in Ethiopia was 12% [5, 6]. In Ethiopia, the earlier studies indicate that one of the basic health problems of the country was childhood diarrheal [4, 6–9].

Several studies have been conducted to examine the determinants of childhood diarrhea in different countries in the world mainly, developing countries including Ethiopia [10–15]. However, in Ethiopia, many types of research have done on institution-based and also are small-geographical areas, focusing on a handful of communities, usually small-sized rural communities [8, 13, 15–18]. This narrowness might be losing efficient results and invalid conclusions as well as recommendations. Besides, the above studies were taking on individual-level analysis by omitting community (cluster) effect. In the individual-level analysis, the independent assumption among clustered observation may not work and the correlations at the individual level may not work at the community (cluster) level and vice versa. For this reason, it is essential to assess the within-cluster and between cluster level variation, and to estimate the true effect of the above-mentioned factors on multilevel determinants of childhood diarrhea in order to implement more effective future health care policies that target specific units at various levels of the hierarchy and there might be a correlation between the higher levels. Therefore, this study aimed to identify both individual and community-level risk factors of childhood diarrheal in Ethiopia using multilevel analysis.

## Methods

### Data source and study design

A study was conducted in Ethiopia, which is situated in the North-Eastern part of Africa, also known as the horn of Africa. This study used the EDHS 2016 dataset which was conducted by the Central Statistical Agency (CSA). This is the fourth national representative survey done at the country level. The stratified multi-stage cluster sampling was used, and it was intended to be representative at the regional and national level in terms of appropriate demographic and health indicators. In the first stage, 645 clusters of enumeration areas (EAs) (202 urban and 443 rural) were identified using probability proportional to the size of EAs. In the second stage, random samples of 18,008 households were selected from all the identified EAs. Lastly, 16,650 households were successfully interviewed, yielding a response rate of 98%. The primary aim of 2016 EDHS was to provide up-to-date information about the key demographic and health indicators. Both men and women aged 15–59 years were interviewed. Data was also collected from mothers or care-takers of live-born infants in the 5 years preceding the date of the interview.

### Study variables

#### Dependent variables

The incidence of childhood diarrhea was the outcome variable of this study.

#### Explanatory variable

The possible variables associated with childhood diarrhea were categorized as individual and community level factors. Those variables were selected by reviews of the literature reviews [6, 8, 9, 11, 14–17, 19–24] and their theoretical justification (Table 1).

**Table 1:** Independent variables and categorization

Variables	Categories
**Individual level factors**	
Age of child (months)	0–23, 24–35, 36–59
Sex of the child	Male; Female
Birth order	First, 2–3, 4 and above
Anemia level	Anemic, not anemic
Child is twin	Single, Multiple
Place of delivery	Home, health facility
Vaccination of child	Yes, No
Size of child at birth	Larger than average, average, smaller than average
Duration of breastfeeding	Ever breastfed, not currently breastfeeding, Never breastfed, Still breastfeeding
Respondent’s age (year)	15–24,25-34,35–49
Highest educational level	No education, Primary, Secondary and above
Family size	≤4, > 4
Wealth index	Poor, Medium, Rich
Current marital status	Separated, Married
Respondent currently working	Not working, Had working
Husband education level	No education, Primary, Secondary and above
Number of children	≤3,> 4
**Community level factors**	
Place of residence	Urban, Rural
Region	Tigray, Afar, Amhara, Oromia, Somali, Beninshangul-Gumuz, SNNPR, Gambela, Harari, Dire Dawa
Toilet facility use	Yes, No
Source of drinking water	Protected, Unprotected

### Data analysis

The secondary data were recorded in SPSS software version 21 and the analysis was done using R software version 3.5.3. Descriptive statistics such as frequencies and percentage were used to summarize the sample data. The 2016 EDHS data which have been collected by stratified multi-stage cluster sampling and data are hierarchical (children nested clusters). The individual children are in general not completely independent of each other [5]. Children from the same cluster will be more similar to each other than children from different clusters. This leads to having too small estimated standard errors that produce spurious ‘significant’ results. Since, clustering effect of the sample should be taken into account during analysis. A multivariable multilevel logistic regression model can account for a lack of independence across levels of nested data [25–27]. For this reason, two-stage multivariable multilevel logistic regression models were used to identify the individual and community level risk factors on childhood diarrhea. The two-stage multivariable multilevel logistic regression model written as follows:

$$ Log\left[\frac{\pi_{ij}}{1-{\pi}_{ij}}\right]={\beta}_0+{\beta}_1{X}_{ij}+{\beta}_2{Z}_{ij}+{u}_j+{e}_{ij} $$

Where, *i* and *j* are the level 1 (individual) and level 2 (community) units, respectively; *X* and *Z* refer to individual and community-level variables, respectively; *π*_*ij*_ is the probability of develop diarrhea for the *i*^*th*^ child in the *j*^*th*^ community; the *β* indicates the fixed coefficients. Whereas, *β*_0_ is the intercept-the effect on the probability of develop diarrhea in the absence of influence of predictors; and *u*_*j*_ showed the random effect (effect of the community on childhood diarrhea for the *j*^*th*^ community and *e*_*ij*_ showed random errors at the individual levels. By assuming each community had different intercept (*β*_0_) and fixed coefficient (*β*), the hierarchical (clustered) data nature and the within and between community variations were taken into account. Four sequential models including the null model were tested: the first model does not include explanatory variables (null model) and only divided the total variance into individual and community component in order to examine the community effect on childhood diarrhea; the second model including only individual-level variables, the third model including only community variables and the fourth model including both the individual- and community-level variables. The results of fixed-effects have been presented in the form of adjusted odds ratios (AORs) with 95% confidence intervals (CIs). The *P*-value ≤0.05 has been considered as statistically significant. The measures of variation (random-effects) included variance, intra cluster correlation (ICC), a proportional change in variance (PCV), and median odds ratio (MOR). The ICC was calculated to evaluate whether the variation in childhood diarrhea is primarily within or between communities. The ICC was computed by the linear threshold according to the formula used by Snijders and Bosker: $$ C=\frac{V_A}{V_A+\raisebox{1ex}{${\pi}^2$}\!\left/ \!\raisebox{-1ex}{$3$}\right.}=\frac{V_A}{V_A+3.29} $$, where *V*_*A*_ is the estimated variance in each model [28]. PCV is used measure the total variation childhood diarrhea attributed to individual-level factors and cluster level factors in the multilevel mode. It was computed using the formula $$ : PCV=\frac{V_A-{V}_B}{V_A} $$, where *V*_*A*_ = variance of the initial model, and *V*_*B*_ = variance of the model with more terms [28]. MOR defined as the median value of the odds ratio between the area at highest risk and the area at the lowest risk when randomly picking out two areas and it depends directly on the community level variance. It was computed using the formula: $$ MOR=\exp \left(\sqrt{2\ast {V}_A\ast 0.6745}\ \right)\approx \exp \left(0.95\sqrt{V_A}\ \right) $$, where *V*_*A*_ is the cluster level variance [27–30]. The existence of multicollinearity was tested by Variance Inflation Factor (VIF) test and variables with value of > 5 were excluded from the model.

### Model fit statistics

The likelihood ratio test (LRT) to assess the goodness-of-fit between two nested models. Model selection was done using Deviance and Akaike’s Information Criterion (AIC). The Deviance and AIC were used to compare various candidate models and the model with the minimum deviance and AIC value is considered as a better fit [31].

## Result

### Prevalence of childhood diarrheal and characteristics of study participants

The overall prevalence of childhood diarrheal in Ethiopia was 12% (95%CI: 11.39, 12.63). The highest diarrheal prevalence (20%) was in Gambela region, and lowest in Addis Ababa (11.5%). The prevalence of diarrhea among females (15.6%) lowers as compared to males (18.4%). Children living in rural areas were reported to be highly exposed to diarrheal diseases as compared with those living in urban areas (17.7% versus 14.4%). Children who never practice breastfeeding were more infected with the diarrheal disease (38.1%). The highest prevalence of diarrheal disease occurred among multiple birth children (29.5%). The occurrence of diarrhea higher among children in children age 36–49 month (27.6%), the smaller size of child at birth(20.7%), mothers age 15–24 years(19.2%), family size less than four(21%) and primary of the school-educated father(18.4%) (Table 2).

**Table 2:** Prevalence of childhood diarrheal by background characteristics Ethiopia, 2016

Background characteristic	Total number of Children		Diarrheal disease status	
	Frequency	Percent	Frequency	Percent
Individual level factors				
Sex of child				
Male	5483	51.5	1009	18.4
Female	5158	48.5	806	15.6
Current age of child				
0–1	4054	38.1	624	15.4
2–3	3856	36.2	438	11.4
4–5	2731	25.7	753	27.6
Anemia level				
Anemic	3589	33.7	613	17.1
Not anemic	7052	66.3	1202	17.0
Birth order number				
First order	2167	20.4	399	18.4
2–4	4661	43.8	779	16.7
Five and above	3813	35.8	637	16.7
Child is twin				
Single birth	10,363	97.4	1733	16.7
Multiple birth	278	2.6	82	29.5
Place of delivery				
Home	6960	65.4	1184	17.0
Health facility	3681	34.6	631	17.1
Child vaccination				
No	8187	76.9	1406	17.2
Yes	2454	23.1	409	16.7
Size of child at birth				
Larger than average	3214	30.2	544	16.9
Average	4419	41.5	649	14.7
Smaller than average	3008	28.3	622	20.7
Duration of breastfeeding				
Ever breastfed, not currently breastfeeding	5814	54.6	950	16.3
Never breastfed	575	5.4	219	38.1
Still breastfeeding	4252	40.0	646	15.2
Maternal age (years)				
15–24	2575	24.2	494	19.2
25–34	6201	58.3	1035	16.7
35–49	1865	17.5	286	15.3
Highest educational level				
No education	6838	64.3	1159	16.9
Primary	2678	25.2	490	18.3
Secondary and above	1125	10.6	166	14.8
Family size				
Less than 4	3007	28.3	632	21.0
Greater than four	7634	71.7	1183	15.5
Wealth index				
Poor	5775	54.3	995	17.2
Middle	1466	13.8	260	17.7
Richer	3400	32.0	560	16.5
Current marital status				
Separated	738	6.9	145	19.6
Married	9903	93.1	1670	16.9
Mother’s occupation				
No	7683	72.2	1279	16.6
Yes	2958	27.8	536	18.1
Husband education level				
No education	4928	46.3	789	16.0
Primary	3220	30.3	594	18.4
Secondary and above	2493	23.4	432	17.3
Number of children				
3 or less	5435	51.1	1056	19.4
4 and above	5206	48.9	759	14.6
Toilet facility				
Yes	5711	53.7	942	16.5
No	4930	46.3	873	17.7
Community level factors				
Place of residence				
Urban	1974	18.6	285	14.4
Rural	8667	81.4	1530	17.7
Region				
Tigray	1033	9.7	172	16.7
Afar	1062	10.0	200	18.8
Amhara	977	9.2	178	18.2
Oromia	1581	14.9	267	16.9
Somali	1505	14.1	203	13.5
Benishangul-Gumuz	879	8.3	140	15.9
Snnpr	1277	12.0	250	19.6
Gambela	714	6.7	144	20.2
Harari	605	5.7	107	17.7
Addis adaba	461	4.3	53	11.5
Dire dawa	547	5.1	101	18.5
Source of drinking water				
Protected	3133	29.4	495	15.8
Unprotected	7508	70.6	1320	17.6

The majority of children (58.3%%) were born to mothers in the age range of 25–34 years. Eighty-one percent of children were born in a rural area and 6.9% of children belong to separated women while the remaining 93.1% were married women. About 97:4% of children were born in singleton, while only about 2.6% have been born in multiple twins. More than half, (54.3%) of children were born from low economic status families, only 32.0% were from rich and (13.8%) were from the medium economic status. About 65.4% of children were delivered at home, while 34.6% were delivered in health facilities. Seventy-two percent of children were from a family size of greater than and only twenty-eight from family size of less than four. The majority, (70.6%) of mothers have used unprotected sources of drinking water (Table 2).

### Factors associated with diarrheal diseases among under-five children

The results of multivariable multilevel logistic regression analysis (fixed effects) were summarized in Table 3. The model comparison result revealed that model IV is a better fit for the data as compared to other reduced models since it has the smallest AIC and Deviance statistic. In this model, the variable sex of a child, age of a child, birth order, types of birth, and size of child at birth, vaccination of child, age of mother, family size, mother working status, number of U5 children, education level of parents, breastfeeding status, residence, region, toilet facility and source of drinking water were significantly associated with childhood diarrhea. The occurrence of diarrhea among female children were 0.840 times (AOR = 0.835; 95% CI: 0.749, 0.931) less likely as compared to male children. Children aged 4–5 years old were 3.17 times (AOR = 3.166; 95% CI: 2.569, 3.900) higher risk of developing diarrhea compared to those whose ages were less than 1 year. Those children whose birth order; 2–4 (AOR = 1.211, 95%CI: 1.026, 1.429) and 5 and above (AOR = 2.210, 95% CI: 1.721, 2.839) were 1.21 and 2.21 times more likely to develop diarrhea than first order. The likelihood of childhood diarrhea was 1.87 times (AOR = 1.871; 95% CI: 1.390, 2.527) higher for children who have multiple birth types as compared to singleton births. Children who were never breastfed were 2.91times (AOR = 2.914; 95%CI: 2.380, 3.567) more likely to develop diarrhea compared to children who were ever breastfed, not currently breastfeeding. Children whose mothers age 25–34 years were 13% (AOR = 0.872; 95% CI: 0.744, 1.022) less likely to develop diarrhea compared to those children whose mothers age 15–24 years. Besides, children with maternal age 35–45 years were 31% (AOR = 0.690; 95% CI: 0.547, 0.871) less likely to develop diarrhea compared to those children whose mothers age 15–24 years. Children with secondary and higher maternal education were 0.77 times (AOR = 0.776; 95% CI: 0.604, 0.996) less likely to experience diarrhea compared to children whose mothers had no education. Similarly, children with secondary and higher maternal education were 1.22 times (AOR = 1.225; 95% CI: 1.011, 1.484) less likely to experience diarrhea compared to children whose mothers had no education The probability of children’s not received any vaccination were 1.21 times (AOR = 1.197; 95% CI: 1.190,1.527) more likely to develop diarrhea as compared to vaccinated children. Children living in a rural area were 1.5 times (AOR = 1.505; 95%CI: 1.233, 1.836) more likely to experience the diarrheal disease as compared to children living in urban areas. Children live in Afar (AOR =1.205: 95%CI: 0.924,1.571), Amhara (AOR 1.493; 95%CI: 1.161,1.921), Benishangul-Gumuz (AOR =1.357; 95% CI: 1.031,1.787), Gambella (AOR = 1.432;95%CI: 1.078,1.902), SNNPR (AOR = 1.609;95%CI: 1.251,2.070) and Dire Dawa (AOR = 1.722; 95%CI: 1.265,2.342) regions were more likely to be infected by diarrhea as compared to children live in Tigray region. Children use unprotected (unimproved) water 1.3 times (AOR: 1.289; 95% CI: 1.060, 1.567) more likely to suffer from diarrhea as compare with a child who uses protected (unimproved) water. Children that were delivered from mothers with no toilet facility were 1.48 times (OR: 1.289; 95% CI: 1.239, 1.759) more vulnerable to diarrhea as compared to infants that were delivered from mother with toilet facility (Table 3).

**Table 3:** Multilevel logistic regression analysis of individual and community level factors associated with childhood diarrhea in Ethiopia, 2016

	Model I AOR (95% CI)	Model II AOR (95% CI)	Model III AOR (95% CI)	Model IV AOR (95% CI)
Individual level factors				
Child characteristics				
Sex of child				
Male		1		1
Female		0.835(0.749, 0.931)*		0.835(0.749,0.931)*
Current age of child				
0–1		1		1
2–3		0.915(0.765, 1.095)		0.901(0.753,1.078)
4–5		3.213(2.610,3.955)		3.166(2.569,3.900)*
Birth order				
first order		1		
2–4		1.214(1.030,1.432)*		1.211(1.026,1.429)*
five and above		2.238(1.743,2.874)		2.210(1.721,2.839)*
Child is twin				
Single birth		1		1
Multiple birth		1.904(1.410,2.572)*		1.871(1.390,2.527)*
Child vaccination				
Yes		1		1
No		1.197(1.012,1.415)*		1.207(1.190,1.527)*
Size of child at birth				
Larger than average				1
Average		0.870(0.761,0.994)*		0.871(0.761,0.996)*
Smaller than average		1.328(1.154,1.529)*		1.303(1.130,1.504)*
Duration of breastfeeding				
Ever breastfed, not currently breastfeeding		1		1
Never breastfed		2.874(2.350,3.516)*		2.914(2.380,3.567)*
Still breastfeeding		1.189(0.998,1.417)		1.151(0.965,1.373)
Maternal age (years)				
15–24		1	1	1
25–34		0.850(0.726,0.994)*		0.872(0.744,1.022)
35–49		0.675(0.537,0.848)*		0.690(0.547,0.871)*
Mother educational level				
No education		1	1	1
Primary		0.981(0.847,1.136)		0.987(0.852,1.145)
Secondary and above		0.715(0.561,0.913)*		0.776(0.604,0.996)*
Family size				
less than 4		1	1	1
greater than four		0.787(0.682,0.909)*		0.783(0.678,0.905)*
Mother’s occupation				
No		1	1	1
Yes		1.136(1.001,1.289)*		1.163(1.023,1.322)*
Father education level				
No education		1		1
Primary		0.574 (0.528, 0.623)*		0.563 (0.502, 0.632)*
Secondary and above		0.403 (0.363, 0.448)*		0.394 (0.364, 0.427)*
Number of children				
3 or less		1		1
4 and above		0.525(0.435,0.634)*		0.526(0.436,0.636)*
Community level factors				
place of residence				
Urban				1
Rural			1.321(1.033,1.689)*	1.505(1.233,1.836)*
Region				
Tigray			1	1
Afar			1.195(1.130, 1.902)*	1.205(0.924,1.571)*
Amhara			1.753(1.370, 2.246)*	1.493(1.161,1.921)*
Oromia			1.212(0.952, 1.543)	1.187(0.928,1.518)
Somali			1.339(1.048, 1.709)*	1.238(0.965,1.587)
Benishangul-Gumuz			1.363(1.043, 1.782)*	1.357(1.031,1.787)*
SNNPR			1.443(1.130, 1.844)*	1.609(1.251,2.070)*
Gambela			1.203(0.911, 1.587)	1.432(1.078,1.902)*
Harari			1.040(0.768, 1.409)	1.086(0.797,1.480)
Addis Adaba			0.597(0.418, 0.851)*	0.737(0.513,1.059)
Dire Dawa			1.839(1.358, 2.489)*	1.722(1.265,2.342)*
Source of drinking water				
Protected			1	
Unprotected			1.414(1.170, 1.709)*	1.289(1.060, 1.567)*
Toilet facility				
Yes				
No			1.454(1.221,1.371)*	1.476(1.239,1.759)*

1 reference category for categorical variable and * reference *P*-value < 0.0001.

### Random effect analysis (measures of variation)

The results of the random effects logistic regression analysis presented in Table 4. The empty model (Model I) indicates that there are communities differences in experiencing diarrhea among children. About 22% of the variance in the odds of childhood diarrhea was attributed to community-level factors (ICC =22%). The MOR (2.56) value of childhood diarrhea was highest in the null model; this reveled that there was variation between communities (clustering) since MOR was 2.56 times higher than the reference (MOR = 1). Moreover, the highest (67.04%) PCV in the full model (model IV), showed that about 67% of the variation in childhood diarrhea across communities was attributed to both individual and community-level factors. The unexplained community variation in childhood diarrhea decreased to MOR of 1.72 when all factors were added to the null model (empty model). This indicates that when all factors are included, the effect of clustering is still statistically significant in the full model (Table 4).

**Table 4:** Measure of variation on individual and community level factor associated with childhood diarrhea in Ethiopia, 2016

Measure of variation	Model I (Null model)	Model II	Model III	Model IV (Full model)
Variance (SE)	0.983(0.041)*	0.424(0.040)*	0.631(.039)	0.326(0.038)*
PCV (%)	Reference	57.07	36.01	67.04
ICC (%)	23.00	11.50	16.09	9.02
MOR	2.56	1.86	2.13	1.72
**Model fit statistics**				
DIC (−2log likelihood)	9647.548	9004.112	9615.004	8967.384
AIC	9651.549	9060.113	9643.004	9049.388

*reference *P*-value < 0.0001

## Discussion

The objective of this study was to identify the risk factors of childhood diarrheal in Ethiopia in Ethiopia using the latest (EDHS-2016) dataset. The prevalence of childhood diarrhea was found to be 12% (95%CI: 11.39, 12.63). The result of this study was higher than the study conducted in Tanzania, 11.9% [32], and Malaysia,4.4% [11]. It was also low as compared to finding in Pader District, northern Uganda, 29.1% [19], Farta District, Northern Ethiopia, 29.9% [6], Gamo Gofa Zone, Southern Ethiopia,27.5% [33]. This may be due to the variation in the sample size of the study, socio-demographic characteristics of the respondents, study period, latrine coverage and utilization, access to clean drinking water.

Gambela region had highest level of diarrhea prevalence (20%) would attribute to life style of residents, where most of the peoples are pastoralist. Due to their migration from place to place in search of pasture and water, pastoralist may not have access to basic health care, protected sources of drinking water, toilet facility and even not much exposed to medias. There is no permanent home for pastoralist and they practice open defecation. Rivers, streams and wells are the main sources of water; thus they are vulnerable to pollution and diarrheal diseases, particularly children who regularly play in the polluted environment. In contrary, the prevalence of diarrhea was lowest (11.5%) in Addis Ababa as compared with other regions in Ethiopia. This result may be attributed to exposure of mothers to mainstream medias, accessible to awareness creations of health facilities and non-governmental organizations where most of NGO’s are resides in the capital city of the country. Mothers who are in Addis Ababa would have a higher chance of being vaccinated and would have a toilet facility and in addition they would have tape water sources. The prevalence of diarrhea among females (15.6%) lowers as compared to males (18.4%). Studies in Ethiopia identified similar patterns of diarrhea among boys than girl [34]. The possible reason might be due to biologic and gender discrimination [34]. It may be that males are more likely to wonder off in unsanitary environment than females. The attribute of this result also needs further investigation.

According to the multilevel logistic analysis indicated, the variation in the childhood diarrhea was attributed to both individual and community level factors. In the final full model (model IV), both individual and community-level factors accounted for about 67.04% of the variation observed for childhood diarrhea. Similar finding also found in Tanzania [35].

Experiencing of diarrhea was significantly associated with sex of child. The occurrences of diarrhea among female children were 0.840 times less likely as compared to male children. This finding is in line with other study findings from Ethiopia [34, 36, 37]. The possible reason might be due to biologic and gender discrimination [34]. The attribute of this result also needs further investigation.

The current age of a child had a significant association with diarrhea occurrence. Children aged 36–59 months were 3.17 times more affected by diarrhea than children age less than 12 months. This is consistent with other studies conducted in Amhara region, Gamo Gofa Zone, Sidama Zone, Ethiopia [6, 8, 33], Tanzania [35], and Northern Uganda [19].

In this study, it was found that there was variation of the diarrhea prevalence rate in terms of child birth order. A higher percentage of diarrhea cases have occurred among the highest birth order. It was found that children whose birth order 4th and above were more likely to be affected by diarrhea than 1st order children. It coincides with the previous studies in which birth order of child increase, the probability of developing childhood diarrhea become increase [9, 20, 36].

Regarding the educational level of parents, children of parents with lower education levels were at high risk of developing diarrhea compared to parents with higher education levels. A similar study in Malaysia found that a 7% higher risk of diarrhea between educated mothers and uneducated mothers and5% higher risk of diarrhea between educated father and uneducated father [11]. Another study was done in Ethiopia [14, 21, 34] and Uganda [19], showed that had higher education levels of parents significant effect in reducing childhood diarrhea. The possible reason might be due to the fact that education is expected to improve household health-care and hygiene practices. Education might assist parents to obtain knowledge about the transmission and prevention mechanism of diarrhea.

In this study, children born from older mothers were less likely to developing diarrhea as compared to children born from young mothers. This finding is in line with studies done in Tanzania [32]. The possible reason could be the discrepancy in awareness related to prevention and treatment of diarrhea attributable to knowledge and information exposure. Older mothers have the potential to gain more information than younger mother about diarrhea from health care providers or other relatives. Households with large family sizes have shown a significant reduction in the risk of childhood diarrhea. This is because member of the family share household duties and mothers have ample time to take care of their children.

The study indicated that the incidence of diarrhea was significantly associated with the duration of breastfeeding. Children who had ever been breastfed had less likely to experience diarrhea as compared to children who had never breasted. This finding had conformity with a study done in Gamo Gofa Zone, Southern Ethiopia [33], Bahir Dar city, Northwest, Ethiopia [4], Medebay Zana district, northwest Ethiopia [13], Ethiopia [34], which showed than children who were partially or not breastfed had a high risk of diarrhea death than exclusively breastfed children. This is because breastfeeding helps to protect child health by preventing contracting contagious diseases including diarrhea.

In the community-level characteristics, the geographical region was a significant predictor of childhood diarrhea. Children living in Afar, Amhara, Benishangul, Gambella, SNNPR and Dire Dawa regions were more likely to be infected by diarrhea as compared to children living in Tigray. This finding is consistent with a study done in Ethiopia [34, 36]. This may be attributed to the large difference in the presence of diarrhea related services including health care, water and sanitation facility and literacy facilities.

Place of residence was a significant predictor of childhood diarrhea in the present study. Children who live in rural areas had higher odds of childhood diarrhea as compared to those children who live in urban. A study in Jamma district, Northeast Ethiopia [21] also showed that the probability of developing diarrhea among rural children higher compared to urban children. This may be due to the people living in rural areas having inaccessibility of adequate facilities such as improved sources of water, sanitation facility, and toilet are some of the consequences. Rural people have not awareness about the transmission, prevention and control of diarrheal disease in comparison to urban people.

This study found that experiencing diarrhea was significantly associated with the availability of toilet facilities in a household. Children that were delivered from mothers with no toilet facility were more vulnerable to diarrhea as compared to children that were delivered from mothers with toilet facilities. This finding is supported by a study done in Gamo Gofa Zone, Southern Ethiopi [33], Jamma district, Northeast Ethiopia [21], Akaki Kality sub-city of Addis Ababa, Ethiopia [38] and North Gondar Zone, Northwest Ethiopia [14, 15], which indicated that children who lived in a household with latrine facility and who defecated in the latrine had lower diarrhea morbidity rate. This may be due that latrine availability reduces fecal environmental pollution and thus decreases the risk of mechanical vectors entering diarrhea-causing organisms to minimize diarrheal disease.

The incidence of diarrhea was significantly associated with a source of water supply. Children from mothers who used unprotected sources of drinking water were more likely to developing of diarrhea as compared to children from mothers who used protected sources of drinking water. This finding is in agreement with a research report from Gamo Gofa Zone, Southern Ethiopia [33], Jamma district, Northeast Ethiopia [21], South Omo Zone, Southern Ethiopia [24] and Malaysia [11], which showed that children use of unprotected water was highly affected by diarrheal. This might be due to the fact that untreated source of drinking water may carry diarrhea-causing pathogens that may lead to diarrhea.

## Conclusion

This study found that both the individual and community factors were associated with childhood diarrhea in Ethiopia. Sex of child, age of the child, birth order, types of birth, size of child at birth, vaccination of child, age of mother, family size, mother working status, number of under-five children, education level of parents, breastfeeding status was significantly associated individual-level factors with childhood diarrhea. Residence, region, toilet facility, and source of drinking water were significantly associated with community level factors with childhood diarrhea. Therefore, integrated child health intervention programs including provisions of toilet facility, access to a clean source of drinking water, educate parents about the importance of breastfeeding and vaccination have to be strongly implemented in order to reduce the high incidence of childhood diarrhea among children.

## Data Availability

The data set was accessed from the Measure DHS website (http://www.measuredhs.com).

## Abbreviations

AIC: Akaike’s information criterion
AOR: Adjusted odds ratio
CI: Confidence intervals
CSA: Central Statistical Agency
DIC: Deviance information criterion
EAs: Enumeration areas
EDHS: Ethiopian demographic and health survey
ICC: Intra-cluster correlation
MOR: Median odds ratio
PCV: Proportional change in variance
SNNPR: Southern Nations, Nationalities, and People Region
WHO: World health organization
